# Aluminum in the United States

**Published:** 1896-06

**Authors:** 


### Aluminum in the United States.

The *Iron Age* estimates the American output of aluminum in 1895 at 850,000 pounds, and believes that the production of the present year will reach the imposing total of 6,000 pounds per day, or over 2,000,000 pounds.

